# Supplementary material for: Bi- and uniciliated ependymal cells define continuous floor-plate-derived tanycytic territories
Source: Nat Commun. 2017 Jan 9;8:13759. doi: 10.1038/ncomms13759 (PMC5477523; doi:10.1038/ncomms13759)
Supplement: Supplementary Information — Supplementary Figures [file ncomms13759-s1.pdf]

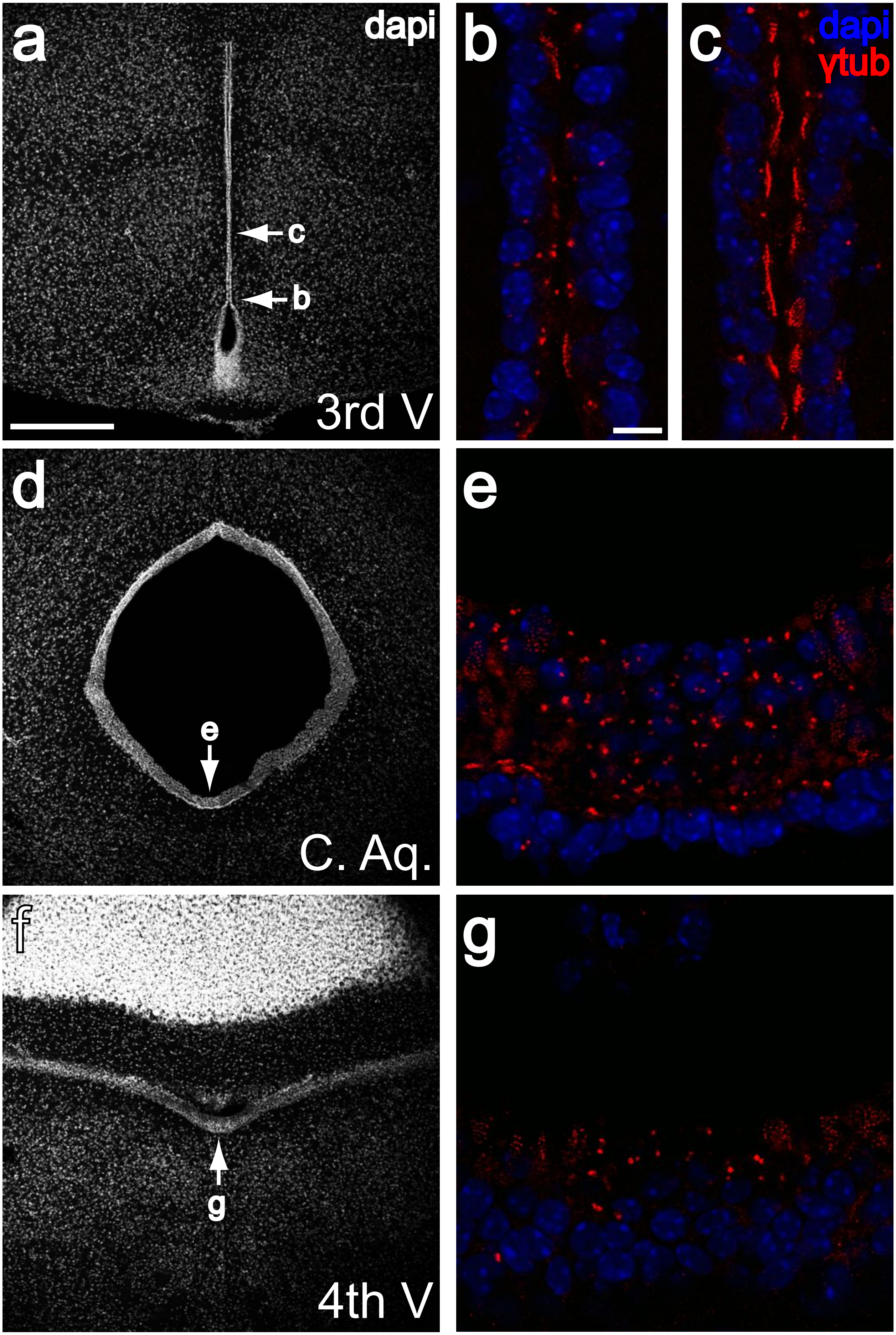

Supplementary Fig. 1. Continuous ventral midline stripe of E2 cells in the 3rd ventricle, cerebral aqueduct, and 4th ventricle. (a,d,f) Representative images of the 3rd ventricle (a), cerebral aqueduct (d), and 4th ventricle (f) from a brain after serial, coronal sectioning revealing that the band of E2 cells was continuous through these regions, as shown in high power images (b,c,e,g) of the sections stained with γ-tubulin and dapi.

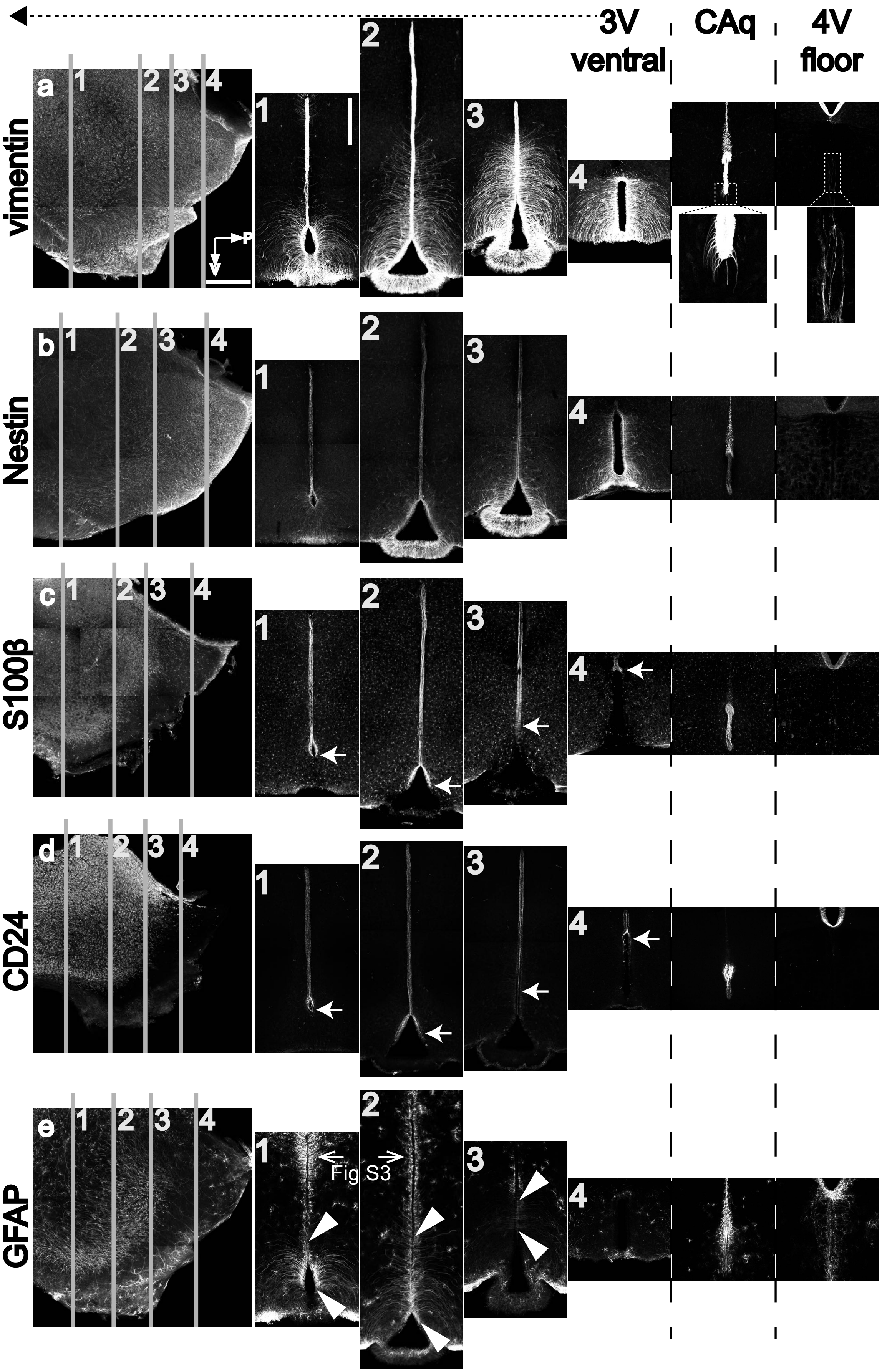

Supplementary Fig. 2. Coronal section correlation of molecular marker expression patterns observed in wholemounts. (a-e) Wholemount perspectives of the 3V infundibular recess immunostained with vimentin (a), nestin (b), S100β (c), CD24 (d), and GFAP (e) as in Figure 3a-e. Vertical gray lines (numbered 1-4) indicate the relative anterior-posterior position of 3V coronal images shown at right, with corresponding numbers. In the far right two columns are the corresponding coronal sections through the cerebral aqueduct and 4V floor. Insets at the far right in (a) show magnified images of vimentin staining in the cerebral aqueduct and 4V floor, revealing long E2 basal processes. Arrows in (c1-4) and (d1-4) indicate the E2-E3 boundary. Arrowheads in (e1-3) delineate the E2 band as seen in coronal sections. Region marked "Fig S3" is shown at higher magnification from a wholemount perspective in that figure. Wholemount and coronal section scale bars, 0.25 mm.

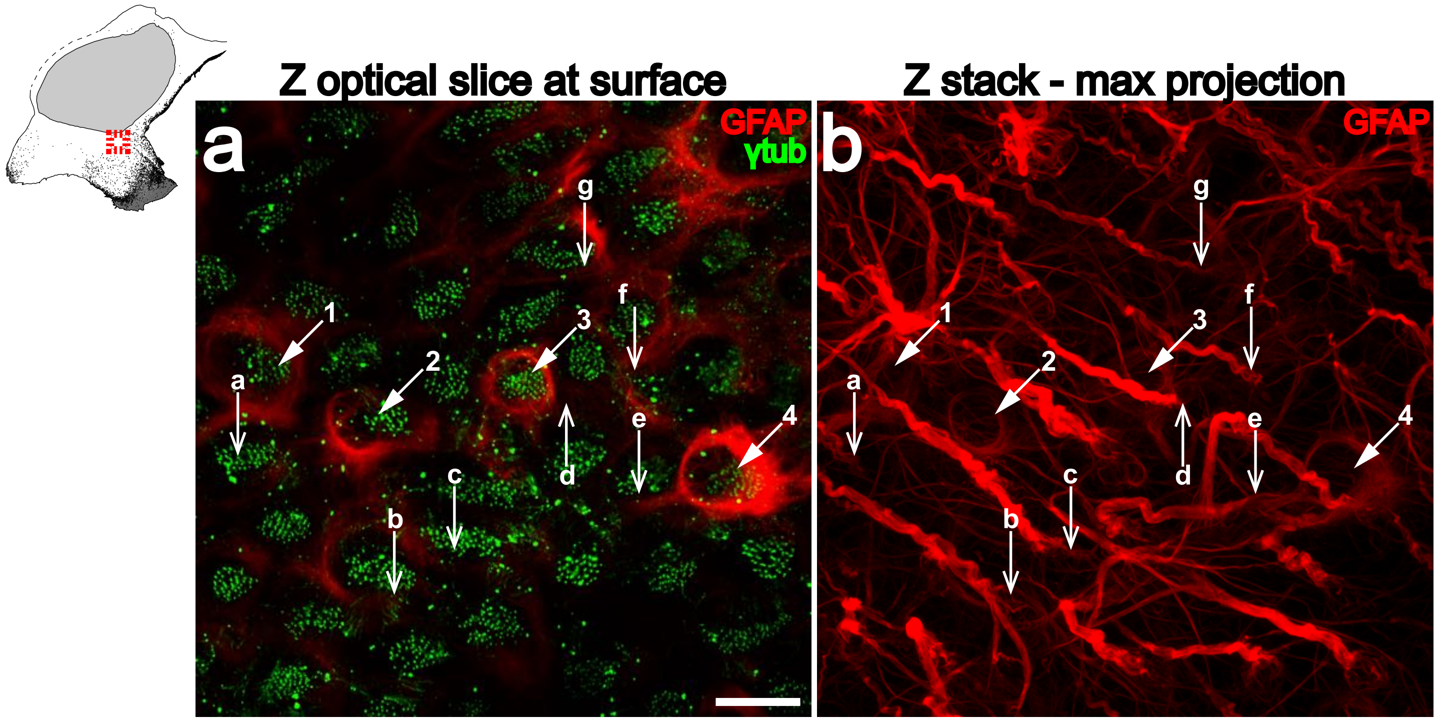

Supplementary Fig. 3. GFAP+ processes dorsal to the E2 band belong to subependymal astrocytes.
In both wholemounts at low magnification and in coronal sections (Fig S2e), we observed abundant cells located dorsal and slightly anterior to the E2 band with moderately-sized processes and intense GFAP+ expression. To determine whether the apical end of these cells contacted the ventricle, we imaged this region in wholemounts stained with GFAP and γ-tubulin. (a) and (b) are images from the same confocal z-stack taken from the boxed region in the map. (a) is a single confocal slice taken at the ependymal surface while (b) is the maximum projection of the entire stack. The apical surface of multiple GFAP+ ependymal cells is indicated with numbers in (a), with the corresponding area also labeled in (b). The letters in (b) indicate the apical end of multiple GFAP+ processes seen in the confocal stack, which do not ultimately make contact with the ventricle, as shown by the absence of these apical surfaces in (a) where they are covered by E1 cells.

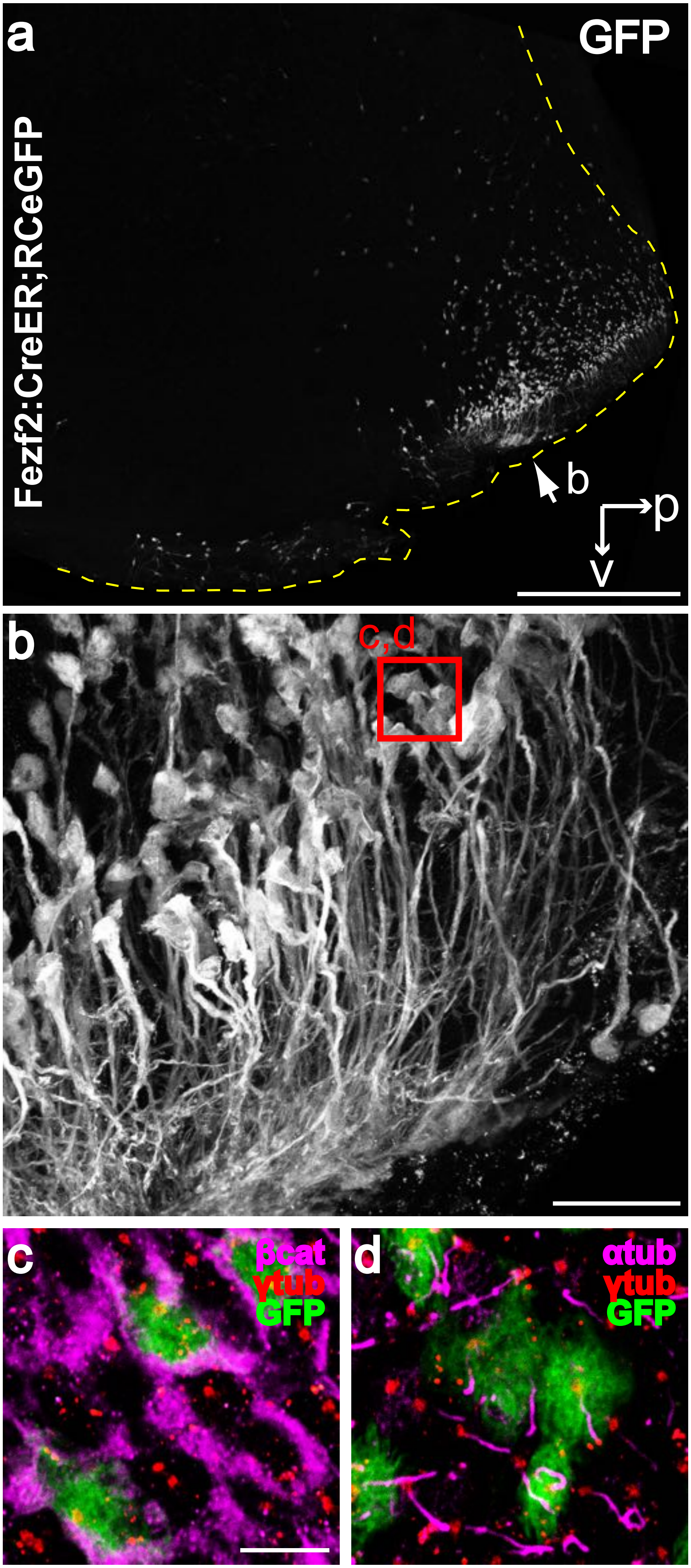

Supplementary Fig. 4. Fezf2-CreER lineage tracing reveals relatively stable position of labeled cells in the E3 territory of the ventral 3V.

(a) Fezf2-CreER mice were crossed to RCeGFP Cre reporter mice; offspring were injected with tamoxifen at P2 and sacrificed at P90 for wholemount preparation and GFP immunostaining. Labeled cells were observed in the E3 territory of the ventral 3V, consistent with the expression pattern in Fezf2-GFP BAC mice and in situ hybridization data from the Allen Brain Atlas. This suggested that in the 3-month period since tamoxifen injection, these labeled E3 cells did not translocate to more dorsal regions outside the E3 territory of the epithelium. (b) At higher magnification, maximally projected confocal z-stacks reveal the typical morphology of GFP+ E3 cells, with their cell body near the ventricular surface and a long basal process extending to the pia. (c)-(d) High magnification confocal images at the ventricular surface of GFP-labeled cells show the characteristic E3 apical specializations, including a small apical surface (delineated by β-catenin [c, magenta]), a single basal body (γ-tubulin [c-d, red]), and a short primary cilium (acetylated α-tubulin [d, magenta]). Scale bar, 0.25 mm (a), 50 um (b), 10 um (c,d).

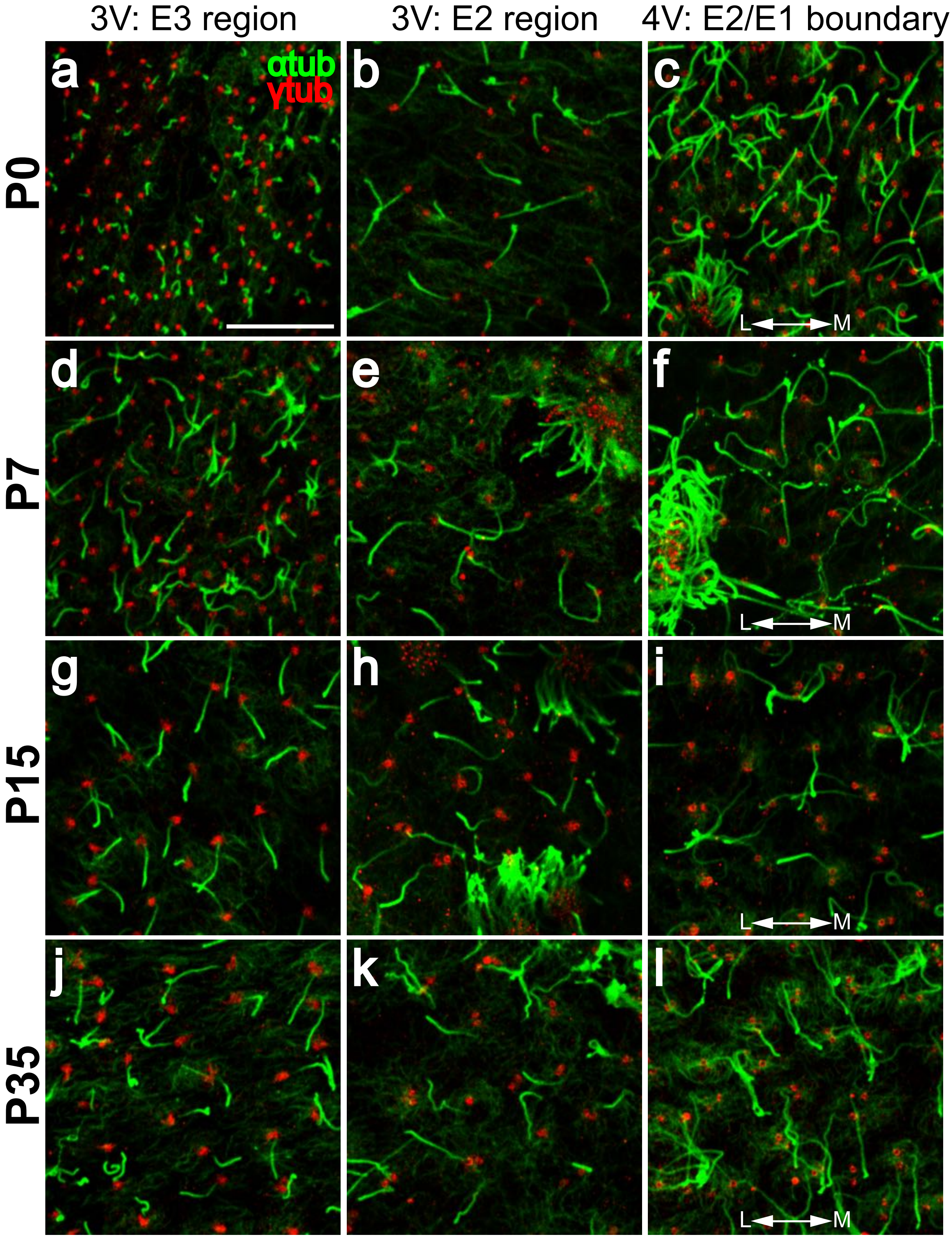

Supplementary Fig. 5. Postnatal development of E2 and E3 cilia.
Apical surface confocal images in the E3 (first column) and E2 (second column) regions of 3V wholemounts and at the E2-E1 boundary (third column) of 4V wholemounts from postnatal ages P0 (a-c), P7 (d-f), P15 (g-i), and P35 (j-l) (corresponding to Figure 5). Wholemounts were labeled with γ-tubulin (red) and acetylated α-tubulin (green) antibodies. At P0, cells in the E3 region had a single, very short cilium consistent with radial glia, while cells in the E2 region were extending single, longer cilia. By P7, cilia in all regions had lengthened, but almost all E2 cells still had only a single cilium. By P15, biciliated E2 cells were observed in the 3V and 4V and the proportion of these biciliated cells, especially in the 4V, increased until P35 when the epithelium reached its adult form. Lateral (L) and Medial (M) directions indicated at the 4V surface. Scale bar, 10 um.

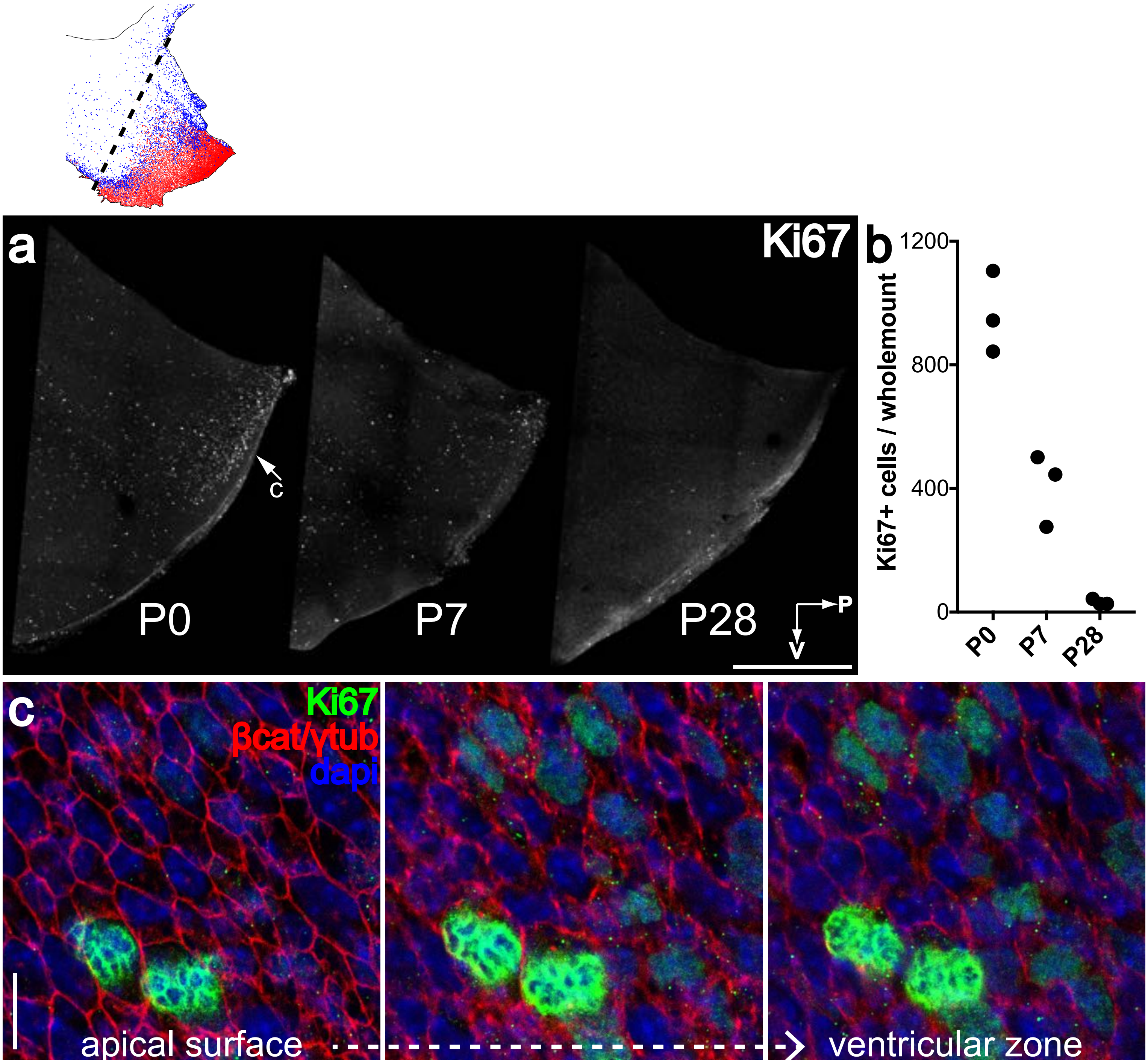

Supplementary Fig. 6. Postnatal decline in proliferation in the ventral 3V.
(a) Postnatal series of wholemounts (3V map above indicates the region shown) at ages P0, P7, and P28 labeled with Ki67 antibodies showed a large number of cycling cells in the caudal ventral 3V at P0 that declined dramatically by P28. (b) Quantification of the number of Ki67+ cells per wholemount (n=3 mice per age, each dot represents 1 mouse). (c) Series of high power confocal images from a z-stack extending from the ventricular surface deeper into the ventricular zone revealed that Ki67+ cells made apical contact with the 3V. Note that both brightly-labeled cells in M phase and more lightly-labeled cells in interphase are observed with apical surfaces contacting the 3V. Scale bar, 0.25 mm (a), 10 um (b).

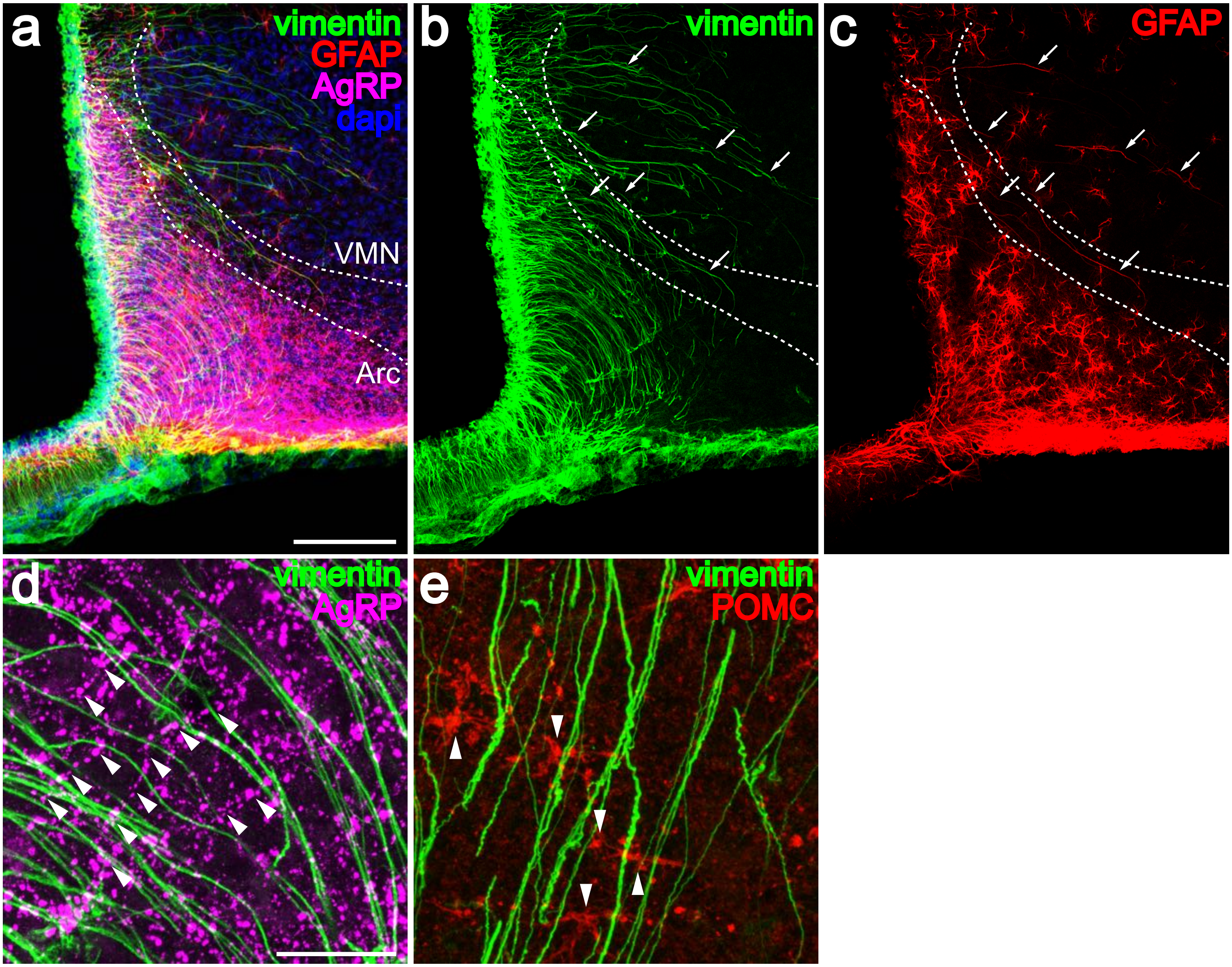

Supplementary Fig. 7. Ventral 3V E3 and E2 cells' basal processes extensively infiltrate the mediobasal hypothalamus and contact neurons.

(a-c) Confocal image of a coronal section through the mediobasal hypothalamus, with dotted lines indicating the boundaries of the arcuate (Arc) and ventromedial nuclei (VMN). Agouti-related peptide (AgRP) neurons occupy the Arc, which is infiltrated by vimentin+GFAP- E3 basal processes. More dorsally, vimentin+GFAP+ E2 processes (arrows) infiltrate the VMN and the lamina between the Arc and VMN. Note that GFAP+ cells in the arcuate nucleus correspond to parenchymal astrocytes. (d-e) Higher magnification confocal images from the Arc reveal extensive contact between vimentin+ E3 processes and AgRP+ neurites (d, magenta) and POMC+ neurons (e, red).
